# Oncofertility Decision Support Resources for Women of Reproductive Age: Systematic Review

**DOI:** 10.2196/12593

**Published:** 2019-06-06

**Authors:** Brittany Speller, Selena Micic, Corinne Daly, Lebei Pi, Tari Little, Nancy N Baxter

**Affiliations:** 1 Department of Surgery St. Michael’s Hospital Toronto, ON Canada; 2 Li Ka Shing Knowledge Institute St. Michael’s Hospital Toronto, ON Canada; 3 Institute of Health Policy, Management, and Evaluation Dalla Lana School of Public Health University of Toronto Toronto, ON Canada

**Keywords:** decision aids, health education materials, fertility, cancer, young women, decision-making, patient education

## Abstract

**Background:**

Cancer treatments have the potential to cause infertility among women of reproductive age. Many cancer patients do not receive sufficient oncofertility information or referrals to reproductive specialists prior to starting cancer treatment. While health care providers cite lack of awareness on the available oncofertility resources, the majority of cancer patients use the internet as a resource to find additional information to supplement discussions with their providers.

**Objective:**

Our aim was to identify and characterize Web-based oncofertility decision aids and health education materials accessible for women of reproductive age with a diagnosis of any cancer.

**Methods:**

We searched five databases and the gray literature for the years 1994-2018. The developer and content information for identified resources was extracted. Each resource underwent a quality assessment.

**Results:**

We identified 31 open access resources including 4 decision aids and 27 health educational materials. The most common fertility preservation options listed in the resources included embryo (31/31, 100%), egg (31, 100%), and ovarian tissue freezing (30, 97%). Notably, approximately one-third (11, 35%) contained references and 5 (16%) had a reading level of grade 8 or below. Resources were of varying quality; two decision aids from Australia and the Netherlands, two booklets from Australia and the United Kingdom, and three websites from Canada and the United States rated as the highest quality.

**Conclusions:**

This comprehensive review characterizes numerous resources available to support patients and providers with oncofertility information, counseling, and decision making. More focus is required to improve the awareness and the access of existing resources among patients and providers. Providers can address patient information needs by leveraging or adapting existing resources to support clinical discussions and their specific patient population.

## Introduction

Many life-saving cancer treatments, including chemotherapy, radiation, and surgery, have the potential to impair reproductive function in women [1-3]. Even if treatment does not directly impact fertility, some cancer treatments are recommended for up to 10 years after diagnosis, delaying pregnancy attempts and resulting in natural fertility declines as patients age [4,5]. As such, women of reproductive age who are diagnosed with cancer have to make a fertility preservation (FP) decision before they begin treatment [3].

The decision to pursue FP is preference-sensitive. There is no “best” option for everyone; rather, the weighting of the risks and benefits of each FP option depend on personal values [6,7]. For optimal decision making, patients need to work in partnership with their health care team to receive fertility information and (when necessary) referrals to reproductive specialists or psychosocial support in a timely manner that promotes understanding of the possible outcomes for different options with consideration of the personal value placed on risks and benefits [8]. This process of shared decision making [9] is particularly important for preference-sensitive decisions, including FP decisions, as it helps ensure that clinical care aligns with patients’ values and preferences [10]. While women of reproductive age want fertility-related information prior to treatment [11,12], in reality many women start cancer treatment without adequate information on treatment-related risks to fertility, potential FP options, or referrals to reproductive specialists [13-15]. The implementation of oncofertility decision aids and health education material early in the clinical pathway is therefore recommended to supplement fertility discussions and assist patients and health care providers in collaborative decision making [8,11,13,16-20].

Decision aids and health education materials could be of great use to women diagnosed with cancer and a valuable tool for providers. However, many providers cite lack of awareness on the available resources as a barrier to information provision and fertility discussions with patients [21-23]. Recently published studies by de Man et al [24] and Mahmoodi et al [25] cataloged and assessed the quality of Web-based fertility health information for women. However, gaps remain in the categorization of available decision aids and health education material and the creation of an inventory of high-quality resources accessible online for providers to use and refer to their patients. Other studies have listed a selection of decision aids and health education materials but were limited to materials in the United States [19,26] and aids with a published evaluation [19,27]. As many patients access Web-based health information as an alternative source of medical information [28], and up to 96% of patients use the internet as a resource for more information [29], there is a need to systematically identify and evaluate existing decision aids and health education materials that are accessible to women and providers. Accordingly, the aim of this systematic review was to identify and characterize Web-based oncofertility decision aids and health education materials accessible for women of reproductive age with a diagnosis of any cancer.

## Methods

### Search Strategy

No protocol was registered for this study. Information specialists conducted a search of MEDLINE, PsycINFO, CINAHL, Cochrane Central and Database of Systematic Reviews, and EMBASE from January 1, 1994, to April 4, 2018, to capture open access decision aids and health education materials available on the Web. Key words and their synonyms were used in the search strategy: [“Fertility” (“Reproductive Techniques,” “Infertility,” “Fertility Preservation,” “Cryopreservation,” “Cryofixation,” “Cryonic Suspension,” “Oocyte Retrieval,” “Oophoropexy”) AND “Cancer” (“Neoplasm,” “Tumor,” “Malignant,” “Oncology,” “Carcinoma,” “Chemotherapy”); OR “Oncofertility”] AND [“Decision Making;” OR “Patient Education.”] (Multimedia Appendix 1). The included articles’ reference lists were manually screened to further identify any relevant publications. The database search was limited to studies on human subjects and publications in English. Consultation with experts in the field of oncology and a Web-based search (Multimedia Appendix 2) allowed for the identification of additional relevant decision aids and health education material not captured in our database search. We searched the Web using the search engine Google [30], as it is the most popular search engine accounting for approximately 75% of Web-based searches [31], and the ClinicalTrials.gov [32] database entering the key phrase “resources for cancer patient’s fertility.” The Google search was run in Canada (Toronto, Ontario) on July 15, 2014, August 17, 2016, and March 13, 2018. We recorded the total number of results and screened the first five pages (approximately 50 website links) as evidence shows most users will not continue their search past the first few pages of search results [33].

### Eligibility and Selection

We included decision aids and health education materials. Decision aids are defined as “evidence-based tools designed to help patients make specific and deliberate choices among healthcare options” [34]. They provide evidence-based information and a personalized focus on treatment options and outcomes to help people clarify their values on the benefits and risks of the available health options to allow for a more informed decision [34,35]. Health education materials “help people understand their diagnosis, treatment and management in general terms, but given their broader perspective, these materials are not focused on decision points” [34]. Inclusion of decision aids and health education materials in this review ensured identification of the diverse resources available through a patient-initiated Web search and those that providers can recommend to patients for supplementary information.

Two reviewers independently screened the websites, publication abstracts, and full texts. Criteria for inclusion included the following: (1) publication/website is in English and describes or is a decision aid or health education material on oncofertility or describes the development and/or evaluation of such a resource, (2) full decision aid or health education material is openly accessible at the time of the search, (3) website contains printable oncofertility information defined by the Patient Education Materials Assessment Tool (PEMAT) as “printed booklets, brochures, and materials that can be printed from websites (eg, PDFs or html text)” [36] or are non-printable websites dedicated to oncofertility, and (4) target audience includes women of reproductive age with a diagnosis of cancer facing an FP decision. We excluded articles that detailed only the development of decision aid components (eg, values clarification methods), survey articles, decision aids or health education materials intended solely for male patients, decision aids or health education materials without open access at the time of the searches, as well as blogs, YouTube videos, forums, and websites from fertility programs/clinics as our search strategy was not designed to capture all clinics globally.

### Data Extraction and Analysis

Two reviewers independently extracted descriptive information into a data extraction table created in Microsoft Excel 2010. Information included author, publication date and date of last update, target population, classification of decision aid or health education material and sections included, number of pages, development country, fertility options before treatment and parenthood options after treatment, and specific content pertaining to fertility (eg, cancer treatments impact on fertility). Analysis of the decision aids and health education materials involved synthesizing descriptive characteristics and tabulating the results.

### Quality Assessment

Since no single quality assessment tool was appropriate for the evaluation of the different decision aids and health education materials identified, we used three separate quality assessment tools based on the type of resource. The International Patient Decision Aid Standards Collaboration (IPDAS) checklist (V.4.0) is internationally approved and recognized as the most credible measure to evaluate the quality of decision aids [37,38]. The modified version used for this review includes 44 items separated into three categories: (1) qualifying as decision aid criteria (6 items), (2) certification criteria (10 items), and (3) quality criteria (28 items), each rated as present or absent [39]. The PEMAT is the main tool used to assess any printable health educational material (eg, brochures, booklets, printable sections of websites) [36]. The PEMAT uses a systematic method to evaluate and compare the understandability and actionability of educational materials. An inventory of 17 characteristics produced an understandability score, and an inventory of 7 items produced an actionability score. Eysenbach et al [40] created the “Seven Quality Domains” for websites that includes 58 quality items most relevant for Web-based health information rated as present or absent, of which 49 items from six domains were applicable to the non-printable websites dedicated to oncofertility identified in this review. Finally, the Flesch-Kincaid readability test was used to determine the grade level of each decision aid and health education material using a readability calculator [41]. For the non-printable websites dedicated to oncofertility, an overall grade level was calculated based on the average readability level of each webpage.

Two reviewers (SM and CD) independently assessed the quality of each decision aid, and two reviewers (BS and TL) independently assessed the quality of each health educational material. The Cohen kappa score was obtained to determine the level of interreviewer agreement [42].

## Results

### Description of Decision Aids and Health Education Materials Identified

**Figure 1:**
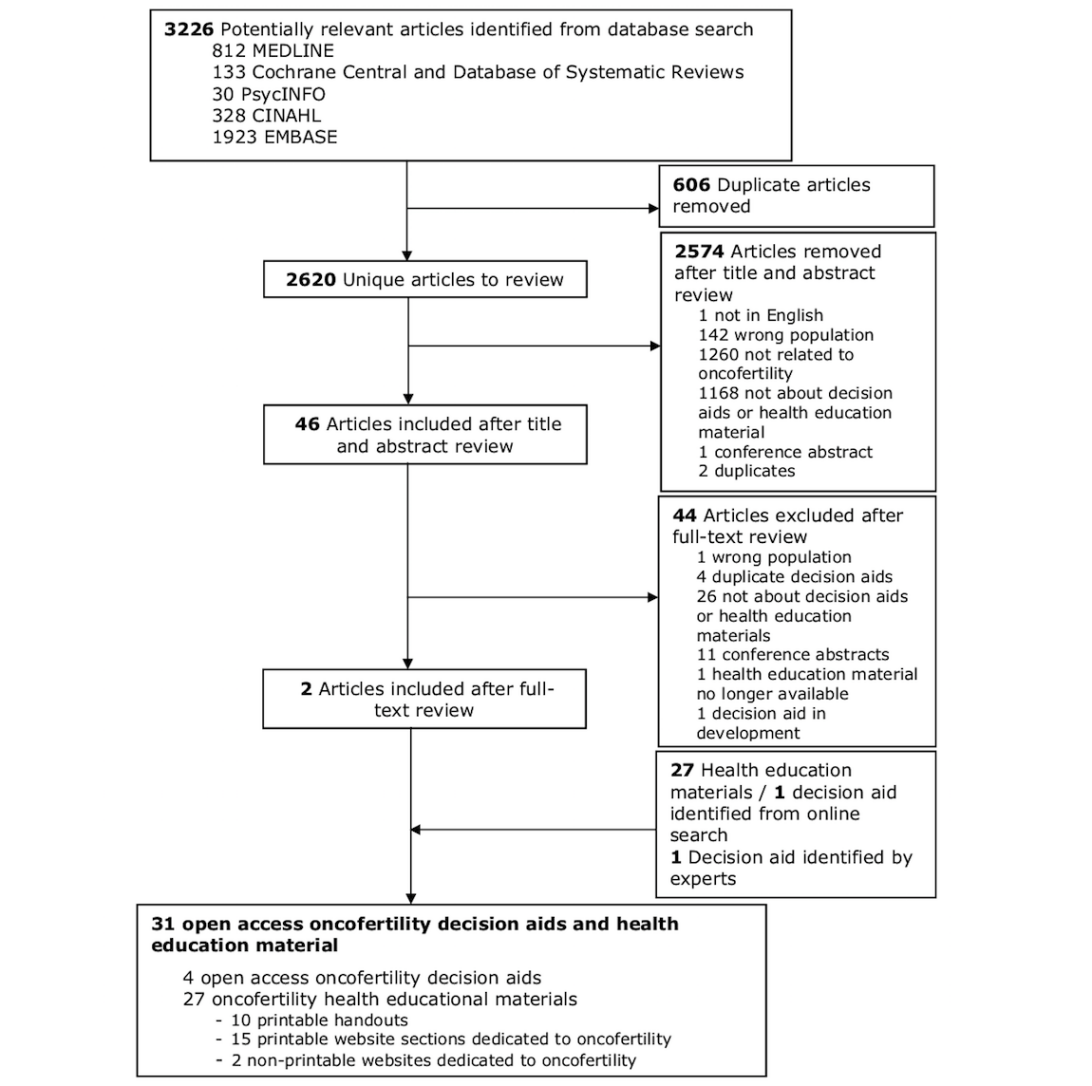
Preferred Reporting Items for Systematic Reviews and Meta-Analysis (PRISMA) flow chart of decision aid and health education material selection.

Figure 1 describes the Preferred Reporting Items for Systematic Reviews and Meta-Analysis (PRISMA) [43] flow chart of systematic database and Web-based study selection resulting in a total of 31 decision aids and health education materials included in this review (Table 1). The database search yielded 2620 unique articles following removal of duplicates. After title and abstract review, 46 articles underwent a full-text review. Two studies describing decision aids met the selection criteria and were included; no additional articles were identified from the reference list review.

The Web-based search in 2014 yielded approximately 11,000,000 results and this increased over twofold in 4 years to approximately 26,600,000 results in the 2018 search. From the Web-based search and consultation with experts in the field of oncology, an additional two decision aids and 27 health education materials were identified. We also identified four decision aids in development, including one in Switzerland by Tschudin et al [44], one in the United Kingdom by the Cancer, Fertility and Me study group and Jones et al [45], one in the United States by Woodard et al [46,47], and one in Germany by Ehrbar et al [48]. These decision aids were not accessible on the Web at the time of the searches and therefore are not included in this review.

This review identified four decision aids categorized as two traditional decision aids (6.5%) and two option grids (6.5%). In 2011, Peate et al developed a decision aid in the form of a booklet for women with early-stage breast cancer in Australia [49]. The Australian decision aid was updated in 2016 and is also being developed into an easily accessible website [50]. In 2013, Garvelink et al developed a Web-based decision aid for women with breast cancer in the Netherlands [51]. In Canada, a shared decision-making fertility option grid was created in 2015 as part of a pan-Canadian study focusing on young breast cancer patients [52]. Finally, a personalizable tool from LIVESTRONG [53] was created that allows patients to input their age, treatment, and cancer type to identify and compare the available options in an option grid format.

An additional 27 health educational materials were identified and categorized as 10 printable handouts (eg, brochures and booklets), 15 printable website sections dedicated to oncofertility (eg, the Canadian Cancer Society contains a section of oncofertility information on their website that is printable), and 2 non-printable interactive websites dedicated to oncofertility. Table 2 outlines the characteristics of all decision aids and health education materials.

**Table 1:** Oncofertility decision aids and health education materials identified (N=31).

Resources identified	n (%)
Decision aids	4 (13)
Health education materials (printable handouts)	10 (32)
Health education materials (printable website sections dedicated to oncofertility)	15 (48)
Health education materials (non-printable website sections dedicated to oncofertility)	2 (7)

**Table 2:** Oncofertility decision aid and health education material description.

Resource		Author	Development group	Year	Type	Number of pages	Language	Sex	Cancer type	Country
**Decision aids**										
	Australian Decision Aid	Peate et al	Academic teaching institution	2011 / 2016	Decision aid booklet	37	English	F^a^	Breast	AUS^b^
	Dutch Decision Aid	Garvelink et al	Academic teaching institution	2013	Decision aid website	26 Web	Dutch	F	Breast	NLD^c^
	SPOKE^d^ Option Grid	Warner et al	Academic hospital	2015	Web-based PDF grid	1	English	F	Breast	CAN^e^
	LIVESTRONG Family Building Option Tool	LIVESTRONG	Non-profit organization	—^f^	Web-based tool	2 Web	English	All	All	USA^g^
**Health educational materials (printable handouts)**										
	ASRM^h^ Fact Sheet	ASRM	Non-profit organization	Revised 2014	Fact sheet	1	English	F	“Female cancers”	USA
	Breast Cancer Care Booklet	Breast Cancer Care	Breast cancer charity	2017	Booklet	36	English	F	Breast	GBR^i^
	Cancer Council Australia Booklet	Cancer Council Australia	Non-government organization	2014/ 2016	Booklet	84	English	All	All	AUS
	Cancer.net	American Society of Clinical Oncology	Non-profit organization	2013	Fact sheet	1	English	All	All	USA
	Cancer Care Fact Sheet	Editor: Lewis, S	National organization	Updated 2017	Fact sheet	2	English	All	All	USA
	Fertile Future Brochure	Fertile Future	Non-profit organization	—	Brochure	6	English / French	All	All	CAN
	LIVESTRONG Booklet	LIVESTRONG	Non-profit organization	2013	Booklet	11	English	All	All	USA
	LLSC^j^ Fertility Facts	LLSC	Voluntary health agency	Revised 2014	Fact sheet	7	English	All	Leukemia / lymphoma	CAN / USA
	Save My Fertility	Oncofertility Consortium	Private research university	2016	Pocket guide	2	English	All	All	USA
	UHN^k^–PMH^l^ Pamphlet	PMH	Teaching hospital	—	Booklet	2	English	F	All	CAN
**Health educational materials (printable website sections dedicated to oncofertility)**										
	ACS^m^	ACS	Voluntary health organization	2017	Educational	2 Web, 16 print	English / Spanish	F	All	USA
	BreastCancer.org	Breast Cancer.org	Non-profit organization	2018	Educational	21 Web	English / Spanish	F	Breast	USA
	CancerPoints	Kantrowitz, M	Cancer information website	—	Educational	1 Web, 7 print	English	All	All	—
	CCS^n^	CCS (ON^o^)	National organization	—	Educational	1 Web, 10 print	English / French	All	All	CAN
	Cleveland Clinic	Cleveland Clinic	Academic hospital	2013	Educational	1 Web, 3 print	English	F	Breast	USA
	Johns Hopkins Medicine	Kolp, L	Private research hospital	—	Educational	5 Web, 12 print	English	All	All	USA
	Mayo Clinic	Mayo Clinic	Non-profit medical practice / research group	2016	Educational	2 Web, 10 print	English / Spanish / Portuguese / Chinese	All	All	USA
	MD Anderson Cancer Center	MD Anderson Cancer Center	Comprehensive cancer center	—	Educational	1 Web, 2 print	English / Spanish / Arabic / Chinese / Turkish	All	All	USA
	MSKCC^p^	MSKCC	Private cancer center	2017	Educational	5 Web, 14 print	English	F	All	USA
	NCCN^q^	NCCN	National organization	—	Educational	1 Web, 4 print	English	All	All	USA
	NCI^r^	NCI	Government health agency	2017	Educational	1 Web, 5 print	English / Spanish	F	All	USA
	NHS^s^	NHS UK	Government health agency	2015	Educational	1 Web, 5 print	English / Google Translate	All	All	GBR
	OncoLink	Vachani, C	Cancer information website	2016	Educational	1 Web, 5 print	English / Spanish	F	All	USA
	WebMD	WebMD	Web-based health publisher	2004	Educational	4 Web, 4 print	English	F	Breast	USA
	YSC^t^	YSC	Non-profit global organization	—	Educational	5 Web, 12 print	English	F	Breast	USA
**Health Educational Materials (non-printable websites dedicated to oncofertility)**										
	Alliance for Fertility Preservation	Alliance for Fertility Preservation	Charitable organization	2015	Educational	42 Web	English	All	All	USA
	Fertile Action	Alice Crisci	Cancer charity	2008	Educational	54 Web	English	F	All	USA

^a^F: female.

^b^AUS: Australia.

^c^NLD: Netherlands.

^d^SPOKE: Surgeon and Patient Oncofertility Knowledge Enhancement.

^e^CAN: Canada.

^f^—: not available.

^g^USA: United States of America.

^h^ASRM: American Society for Reproductive Medicine.

^i^GBR: United Kingdom of Great Britain and Northern Ireland.

^j^LLSC: The Leukemia & Lymphoma Society of Canada.

^k^UHN: University Health Network.

^l^PMH: Princess Margaret Hospital.

^m^ACS: American Cancer Society.

^n^CCS: Canadian Cancer Society.

^o^ON: Ontario.

^p^MSKCC: Memorial Sloan Kettering Cancer Center.

^q^NCCN: National Comprehension Cancer Network.

^r^NCI: National Cancer Institute.

^s^NHS: National Health Service.

^t^YSC: Young Survival Coalition.

### Fertility and Parenthood Options Presented in Decision Aids and Health Education Materials

All resources identified provided information on embryo and egg freezing. Most resources provided information on ovarian tissue freezing (30/31, 97%) and many provided information on ovarian suppression (23/31, 74%). Less than half of resources provided information on other FP options including ovarian transposition (13/31, 42%), fertility-sparing surgery (12/31, 39%), ovarian shielding (6/31, 19%), and in vitro maturation (5/31, 16%). The Australian and Dutch decision aids as well as the PMH pamphlet, MSKCC website, Breast Cancer Care booklet, and Cancer Council Australia booklet (6/31, 19%) were the only resources to included information on the option of not pursuing FP or “wait and see.” Nine resources (29%) provided no additional information on parenthood options after cancer treatment. The most commonly described parenthood options after treatment included egg donation (17/31, 55%), surrogacy (17/31, 55%), adoption (15/31, 48%), natural conception/having fertility testing completed (14/31, 45%), and embryo donation (13/31, 42%). Few resources listed no more children (6/31, 19%) or foster parenting (2/31, 6%) as parenthood options after cancer treatment. Multimedia Appendix 3 presents all fertility options listed in each resource.

### Content and Sections in Decision Aids and Health Education Materials

The Australian decision aid and Cancer Council Australia booklet were the most comprehensive resources covering a range of topics and included sections. These resources also contained the most pages, with 37 and 84 pages of content respectively. Only the decision aids from Australia and the Netherlands contained explicit values clarification methods. The values clarification method in the Australian decision aid is a personal worksheet with questions and a pros/cons list to identify the drawbacks and advantages for each fertility option [49]. The Dutch decision aid includes a 5-point scale where patients indicate their preference towards a fertility option by sliding the scale from very negative to very positive [51].

Few resources contained information on fertility in women (10/31, 32%), with more focusing on infertility in women (13/31, 42%). Most resources included information on cancer treatments (22/31, 71%), an explanation on how the treatment impacts fertility (25/31, 81%) and fertility outcomes after treatment (eg, reduced fertility, early menopause or immediate menopause) (21/31, 68%). Many resources also listed sources for patients to access more information (23/31, 74%). Finally, 11 resources (35%) contained references detailing the sources of evidence and 7 resources (23%) had a glossary of medical terms. Multimedia Appendix 4 lists the content for each decision support resource identified.

### Quality Assessment of Decision Aids and Health Education Materials

Each resource underwent a quality assessment (Multimedia Appendix 5). The Cohen kappa score indicated substantial interrater agreement for all reviewers (0.75 kappa score) [42]. Table 3 outlines the highest rated decision aids and health education materials (printable and non-printable) based on the specific quality assessment used.

The decision aid quality assessment revealed that the Australian and Dutch decision aids met all the qualifying criteria, while the two option grids met most qualifying criteria (5/6, 83%). The Dutch decision aid met all certification criteria, while the Australian decision aid and Canadian option grid met most applicable certification criteria, (5/6, 83% and 4/6, 67% respectively), and the LIVESTRONG option grid met only one certification criteria (1/6, 17%). All decision aids met at minimum three of the applicable quality criteria, including the ability to compare features of available options, inclusion of outcome probabilities, and the event rates for the outcome probabilities. The Australian and Dutch decision aids met the most quality criteria, (19/23, 83% and 20/23, 87%, respectively), when compared to the option grids.

**Table 3:** High-quality oncofertility decision aids and health education materials based on International Patient Decision Aid Standards Collaboration (IPDAS), Patient Education Materials Assessment Tool (PEMAT), and Seven Quality Domains.

Resource		Quality assessment tool	Quality assessment rating
**Decision aid**			
	Australian Decision Aid	IPDAS	Qualifying criteria: 100% Certification criteria: 83% Quality criteria: 83%
	Dutch Decision Aid	IPDAS	Qualifying criteria: 100% Certification criteria: 100% Quality criteria: 87%
**Health educational materials (printable handouts)**			
	Breast Cancer Care Booklet	PEMAT	Understandability score: 87% Actionability score: 80%
	Cancer Council Australia Booklet	PEMAT	Understandability score: 94% Actionability score: 80%
**Health educational materials (printable website sections dedicated to oncofertility)**			
	Canadian Cancer Society	PEMAT	Understandability score: 83% Actionability score: 80%
	Memorial Sloan Kettering Cancer Center (MSKCC)	PEMAT	Understandability score: 80% Actionability score: 80%
**Health educational materials (non-printable websites dedicated to oncofertility)**			
	Alliance for Fertility Preservation	Seven Quality Domains	38/49 quality characteristics (76%)

Using PEMAT, five of the printable handouts and printable website sections dedicated to oncofertility shared the greatest actionability score (4/5, 80%) (ie, material was the most actionable for patients), including the Cancer Council Australia booklet, the Leukemia and Lymphoma Society factsheet, the Breast Cancer Care booklet, and the Breastcancer.org, Canadian Cancer Society, MD Anderson Cancer Center, and MSKCC websites. Six materials rated 80% or above on understandability (ie, material was more understandable for patients), including the Cancer Council Australia booklet (16/17, 94%), American Cancer Society (13/15, 87%), Breast Cancer Care booklet (13/15, 87%), Canadian Cancer Society website (10/12, 83%), the MSKCC website (12/15, 80%), and the National Cancer Institute website (12/15, 80%). More than half (64%) of the printable handouts and printable website sections dedicated to oncofertility scored 50% or below on the actionability, and 52% of these resources scored below 70% on the understandability. However, all materials used the active voice for most sentences, did not expect users to complete any calculations, did not contain material that distracted from the resources purpose, and presented the information in a logical sequence.

The interactive oncofertility dedicated websites had variable quality, meeting between 23 (47%) and 38 (76%) of the 49 possible criteria. Both websites contained technical elements such as information on the ownership of the site, clear statement about their objectives and target audience, transparency on funding, compliance with advertising rules, and geographic location of the site. Additionally, each website contained design elements such as scroll bars, subheadings and grouping of information, a menu with listings, proper layout and typography, and correct presentation of content when viewed in a partial webpage window. For readability and usability, the websites had appropriate sentence construction, use of active voice for most sentences, and road signs to indicate next/previous topics, minimal downloading time, appropriate functionality to support content, and ease of navigation in finding the desired content. However, some aspects that neither website displayed included the date of creation/last update/technical maintenance, message alert when leaving the secure site, clear statement about the editorial review process, hierarchy of evidence clearly displayed, and interactive learning tools (eg, Web-based quiz).

Only five (16%) of the decision aids and health education materials were assessed at a reading level of grade 8 or below. All other resources ranged from a grade 8 to grade 12 and above readability level (Multimedia Appendix 6).

## Discussion

### Principal Considerations

This review identified and characterized 31 open access decision aids and health education materials of varying quality for use by women of reproductive age diagnosed with cancer and their providers. Of the identified resources, two decision aids from Australia [49] and the Netherlands [51], two printable handouts from the United Kingdom [54] and Australia [55], and two websites from Canada [56] and the United States [57,58] rated as the highest quality. This review adds multiple new decision aids and health education materials for women of reproductive age with cancer to the three Web-based health education materials from the United States identified by Kelvin et al in 2012 [26], and the one decision aid for early stage breast cancer patients (Australian decision aid [49]) identified in a 2016 review by Zdenkowski et al [27]. Zdenkowski et al described a gap in oncofertility decision aids for young breast cancer patients, and this review revealed that decision aids are now available or under development for cancer patients in Canada, the Netherlands, Germany, Switzerland, the United States, and the United Kingdom. This review also expands on two recent reviews by de Man et al [24] and Mahmoodi et al [25] and further characterized the oncofertility decision aids and health education materials available for women of reproductive age diagnosed with cancer, extended the categorization and quality analysis by type of resource, and includes a quick reference list that practitioners can use to identify high-quality decision aids and health education materials to supplement fertility discussions and recommend to their patients. The creation and use of resources as an adjunct to fertility discussions with providers is strongly supported in the literature [8,19]. Additionally, this review highlighted the increased attention surrounding the topic of oncofertility in cancer patients as the search engine results more than doubled from 2014 to 2018.

While the number of resources has increased, this review found the quality of these resources could be enhanced. While more information is of benefit to patients and providers, developers should adhere to best practices, such as the IPDAS [35] when creating decision aids to ensure resources are high quality and usable by the target population and the Standards for Universal reporting of patient Decision Aid Evaluation (SUNDAE) checklist [59] when reporting on evaluations of decision aids. Additionally, only the IPDAS checklist evaluated if the decision aids underwent field testing with patients and providers. As the health educational materials were identified through the Web-based search, it was unclear if there had been any field testing of these materials with target users. Field testing is recommended by the IPDAS to ensure the information in the resource resonates with and is understood by the population of interest and does not cause any bias in decision making [60].

The Australian decision aid by Peate et al [49] and booklet by Cancer Council Australia [55] were the most comprehensive and detailed resources identified in our search. However, both resources were long, highlighting the tradeoff between comprehensiveness and ease of use in clinic for patients and providers. Longer resources may be more useful as a take-home resource since limited clinic time may result in the inability for patients and providers to fully review the resource and have in-depth fertility discussions. Yet, a challenge with comprehensive resources used by patients independently outside of clinic is the inability to guarantee that shared decision making occurs in follow-up consultations [61]. In comparison, resources such as the Canadian option grid were specifically designed to be used as a concise in-clinic shared decision-making tool with patients and providers. However, effective use of these in-clinic resources requires the active involvement and engagement of providers [62]. To ensure continued and proper use of in-clinic resources, providers must agree on need for the resource, use the resource in clinic regularly, and administer the resource effectively to promote shared decision making with patients [62-64].

Women of reproductive age want fertility information and desire participation in discussions around FP prior to starting fertility-risking cancer treatment [65]. The risk of infertility from cancer treatment is of such importance to women that it can impact treatment decision making [66]. As such, patients’ information needs are also important for providers to consider when deciding on the appropriate resource to provide as an adjunct to discussions. Some patients may benefit from shorter resources (eg, option grid or fact sheet) and more in-clinic shared decision making, whereas others may prefer more comprehensive resources that provide information on fertility, exposure to all available FP and parenthood options, and assistance in decision making. Additionally, some patients may benefit from both types of resources in clinic and to review independently or with their support person(s). This review identified a wide range of easily accessible resources, alleviating the barrier of lack of awareness on the available resources cited by providers [21-23]. Providers should promote the high quality and applicable resources to interested patients based on their identified information needs. Resource developers can also modify existing resources to improve their quality and meet the needs of their patient population. To enable use of the resources, developers should create a dissemination and education plan that is aligned with patients’ needs and providers’ practices to ensure accessibility and continued use [67].

Through the exploration of Web-based sources, the review was strengthened by the discovery of decision aids in development and resources not identified in previous reviews [19,27,68]. This review also included various resources created by academic centers, non-profit organizations, and charities for cancer patients accessible through a search engine query. While this review excluded resources designed solely for men, it is important to highlight that male-specific resources are also necessary to identify and evaluate. However, due to the differences in infertility risks and FP options between men and women [69], male resources should be characterized and evaluated in a separate review [70]. Only open access and English language resources were included. As such, resources not identified using the key search terms and phrases at the time of the search, resources in another language, or resources only accessible when logged on to an organization’s network server may have been missed in this review. The characteristics of the resources including the content and the fertility options presented in this review may change as developers update them to reflect advances in the field of oncofertility.

We also conducted the Web-based search using one search engine (Google) in one location (Toronto, Ontario). Although different results may have been obtained with other search engines and in other geographic locations, the search was conducted at three different time points capturing search engine index changes. Additionally, the review of approximately 50 websites during each search ensured a broad range of potentially eligible websites and aimed to replicate the searching strategy of a patient recently diagnosed with cancer. Our search did not include fertility clinic information as the search strategy was not designed to capture fertility clinics globally and a targeted search of fertility clinic information was out of scope for this review.

### Practical Implications

This review allowed for the comparison and quality assessment of decision aids and health education materials potentially accessed by women of reproductive age with a diagnosis of cancer or used by providers as an adjunct to clinical discussions. Applicable resources that align with the clinical population, local context, and patient information needs can be identified from this review. As such, we need to focus on enhancing the awareness and the access of these resources to ensure use and promotion of high-quality resources to patients who desire more information before fertility decision making and cancer treatment. The identified decision aids and health education materials can also be modified to enhance their quality and to meet the local needs of a clinic and patient population.

### Conclusion

Fertility preservation prior to cancer treatment is an important topic of discussion for women of reproductive age, and resources can help facilitate patient-provider discussions prior to fertility-risking treatment. This review identified 31 oncofertility decision aids and health education materials that are publicly available. The quality assessments revealed the resources are of varying quality, which indicates that there is room for improvement for many of these resources. As further resources are developed to fill an information gap, developers should adhere to patient education best practices during development to ensure a high-quality tool. Field testing should also be completed by stakeholders of the resource prior to publication of the content on the Web.

## Appendix

Multimedia Appendix 1 Search strategy (MEDLINE) used to retrieve records from January 1994 to April 2018 reporting oncofertility decision aids and health education materials.

Multimedia Appendix 2 Online sources searched.

Multimedia Appendix 3 Fertility and parenthood options in oncofertility decision aids and health education materials.

Multimedia Appendix 4 Content and sections in the oncofertility decision aids and health education materials.

Multimedia Appendix 5 Quality assessments of the oncofertility decision aids and health education materials.

Multimedia Appendix 6 Readability level of the oncofertility decision aids and health education materials using the Flesch-Kincaid Grade Level test.

## Abbreviations

FP: fertility preservation
IPDAS: International Patient Decision Aid Standards Collaboration
MSKCC: Memorial Sloan Kettering Cancer Center
PEMAT: Patient Education Materials Assessment Tool
PRISMA: Preferred Reporting Items for Systematic Reviews and Meta-Analysis
SUNDAE: Standards for Universal reporting of patient Decision Aid Evaluation
