# Supplementary material for: Oncofertility Decision Support Resources for Women of Reproductive Age: Systematic Review
Source: JMIR Cancer. 2019 Jun 6;5(1):e12593. doi: 10.2196/12593 (PMC6592478; doi:10.2196/12593)
Supplement: Multimedia Appendix 1 [file cancer_v5i1e12593_app1.pdf]

Multimedia Appendix 1. Search strategy (MEDLINE) used to retrieve records from January 1994 to April 2018 reporting oncofertility decision aids and health education materials

1. exp Reproductive Techniques, Assisted/
2. exp Fertility/
3. exp Infertility, Female/
4. (fertility adj5 (preserv* or conserve* or conservation or sparing or saving)).mp.
5. ((preserv* or conserve* or conservation) adj5 reproduct*).mp.
6. exp Cryopreservation/
7. cryopreservation*.mp.
8. cryofixation.mp.
9. (cryonic adj suspension*).mp.
10. (oocyte adj4 retrieval*).mp.
11. (oophoropexy or oophoropexies).mp.
12. ((fertility or infertility) adj5 (specialist* or consultant* or counselor* or counsellor*)).mp.
13. 1 or 2 or 3 or 4 or 5 or 6 or 7 or 8 or 9 or 10 or 11 or 12
14. exp Neoplasms/
15. neoplas*.mp.
16. cancer*.mp.
17. (tumor* or tumour*).mp.
18. malignan*.mp.
19. (oncology or oncologic* or oncologist*).mp.
20. carcinoma*.mp.
21. exp Consolidation Chemotherapy/
22. exp Induction Chemotherapy/
23. exp Maintenance Chemotherapy/
24. exp Chemotherapy, Cancer, Regional Perfusion/
25. exp Chemotherapy, Adjuvant/
26. Antineoplastic Agents/
27. exp Angiogenesis Inhibitors/
28. exp Antibiotics, Antineoplastic/
29. exp Anticarcinogenic Agents/
30. exp Antimetabolites, Antineoplastic/
31. Antimitotic Agents/
32. exp Antineoplastic Agents, Alkylating/
33. exp Antineoplastic Agents, Hormonal/
34. exp Antineoplastic Agents, Phytogenic/
35. exp Myeloablative Agonists/
36. exp Topoisomerase Inhibitors/
37. exp Chemoembolization, Therapeutic/
38. chemotherap*.mp.
39. antineoplastic*.mp.
40. (antitumor or antitumour).mp.
41. anticancer.mp.
42. alkylating.mp.

43. melphalan.mp.
44. cyclophosphamide.mp.
45. cisplatin.mp.
46. matulane.mp.
47. methotrexate.mp.
48. vincristine.mp.
49. procarbazine.mp.
50. ifosfamide.mp.
51. busulfan.mp.
52. chlorambucil.mp.
53. hlormethine.mp.
54. doxorubicin.mp.
55. carboplatin.mp.
56. adenocarcinoma*.mp.
57. (brca or (breast adj4 (adenocarcinoma* or cancer* or carcinoma* or metasta* or neoplas* or tumo?r*))).ti,ab.
58. lymphoma*.mp.
59. (leukemia* or leukaemia*).mp.
60. ((brain or intracranial) adj4 (cancer* or carcinoma* or metasta* or neoplasm* or tumo?r*)).ti,ab.
61. ((cervix or cervical) adj4 (adenocarcinoma* or cancer* or carcinoma* or metasta* or neoplas* or tumo?r*)).ti,ab.
62. ((uterus or uterine or endometri*) adj4 (adenocarcinoma* or cancer* or carcinoma* or metasta* or neoplas* or tumo?r*)).ti,ab.
63. ((ovary or ovarian) adj4 (cancer* or carcinoma* or metasta* or neoplas* or tumo?r*)).ti,ab.
64. 14 or 15 or 16 or 17 or 18 or 19 or 20 or 21 or 22 or 23 or 24 or 25 or 26 or 27 or 28 or 29 or 30 or 31 or 32 or 33 or 34 or 35 or 36 or 37 or 38 or 39 or 40 or 41 or 42 or 43 or 44 or 45 or 46 or 47 or 48 or 49 or 50 or 51 or 52 or 53 or 54 or 55 or 56 or 57 or 58 or 59 or 60 or 61 or 62 or 63
65. 13 and 64
66. oncofertility.mp.
67. 65 or 66
68. exp Decision Making/
69. (decision* or decide* or deciding).mp.
70. (choice* or choose).mp.
71. Patient Education as Topic/
72. educat*.mp.
73. 68 or 69 or 70 or 71
74. 67 and 73
75. limit 74 to (english language and yr="1994 -Current")
76. 75 not (exp animals/ not humans/)
77. remove duplicates from 76
