# Supplementary material for: Oncofertility Decision Support Resources for Women of Reproductive Age: Systematic Review
Source: JMIR Cancer. 2019 Jun 6;5(1):e12593. doi: 10.2196/12593 (PMC6592478; doi:10.2196/12593)
Supplement: Multimedia Appendix 2 [file cancer_v5i1e12593_app2.pdf]

Multimedia Appendix 2. Web-based sources searched

- American Association for Cancer Research
- Advanced Fertility Care
- American Cancer Society
- American Society for Reproductive Medicine
- American Society of Clinical Oncology
- Barbados Fertility Clinic
- BC Cancer Agency
- Breast Cancer Care
- BreastCancer.org
- Canadian Cancer Society
- Cancer Council Australia
- Cancer Guidelines Wiki
- Cancer Knowledge Network
- Cancer Points
- CancerCare
- CancerConnect.com
- Carolina Conceptions
- Cleveland Clinic
- ClinicalTrials.gov
- Coastal Fertility Medical Center
- ColumbiaDoctors - Center for Women's Reproductive Care
- Fertile Action
- Fertile Future
- Fertility Associates of Memphis
- Fertility Authority
- Fertility C.A.R.E.
- Fertility Centers of Illinois
- Fertility Matters
- Fertility Preservation in Pittsburgh
- Fertility Today Magazine
- Fertility Within Reach
- Flinders Fertility
- Genetics & IVF Institute
- Jewish General Hospital
- John Hopkins Medicine
- JourneyForward
- Livestrong Foundation
- Macmillan, Cancer Support
- Maryland Department of Health
- Mayo Foundation for Medical Education and Research
- MD Anderson Cancer Center
- Memorial Sloan Kettering Cancer Center
- MyOncofertility.org
- National Cancer Institute
- National Comprehensive Cancer Network
- National Health Service (United Kingdom)
- Northwell Health Fertility
- OncoLink
- Pacific Fertility Center
- Patient Resource
- Reproductivefacts.org
- ReproTech Limited
- RESOLVE: The National Infertility Association
- Rethink Breast Cancer
- Roswell Park Cancer Institute
- SaveMyFertility.org
- Sexual Health & Fertility curated at the Resource and Learning Center
- Sher Institutes for Reproductive Medicine
- Stanford Medicine
- Stupid Cancer
- Teenage and Young Adults with Cancer (TYAC)
- Texas Fertility Center
- The Alliance for Fertility Preservation
- The Center for Reproductive Medicine
- The Hospital for Sick Children
- The Infertility Center of St. Louis
- The Infertility Voice
- The Leukemia and Lymphoma Society
- The Official Journal of the National Comprehensive Cancer Network
- The Oncofertility Consortium at Northwestern University
- The Ottawa Hospital Research Institute, Decision Aid Library Inventory
- The SAMFund
- The University of Pennsylvania - Fertility Care
- U.S. Department of Health & Human Services - Agency for Healthcare Research & Quality
- University Health Network - Princess Margaret Cancer Centre
- University of California San Diego Moores Cancer Center
- University of California, San Francisco, Center for Reproductive Health
- University of Colorado - Advanced Reproductive Medicine
- University of Florida Health Cancer Center
- University of Michigan Health System - Comprehensive Cancer Center
- University of Utah - Huntsman Cancer Institute
- USC Fertility Organization
- WebMD
- Westmead Fertility Center
- Young Survivor Coalition
