# Supplementary material for: Oncofertility Decision Support Resources for Women of Reproductive Age: Systematic Review
Source: JMIR Cancer. 2019 Jun 6;5(1):e12593. doi: 10.2196/12593 (PMC6592478; doi:10.2196/12593)
Supplement: Multimedia Appendix 3 [file cancer_v5i1e12593_app3.pdf]

Multimedia Appendix 3. Fertility and parenthood options in oncofertility decision aids and health education materials

Resource Name	Fertility Options Before Treatment									Parenthood Options After Treatment						
	Wait and see	Egg freezing	Embryo freezing	Ovarian tissue freezing	Ovarian suppression	Ovarian transposition	Ovarian shielding	Fertility-sparing surgery	*In vitro* maturation	No more children	Egg donation	Embryo donation	Surrogacy	Adoption	Foster parenting	Natural conception/ Fertility testing
Decision Aids																
Australian Decision Aid	✓	✓	✓	✓	✓	–	–	–	–	✓	✓	✓	✓	✓	–	–
Dutch Decision Aid	✓	✓	✓	✓	–	–	–	–	–	✓	✓	–	–	✓	✓	–
SPOKE Option Grid	–	✓	✓	✓	✓	✓	–	–	–	–	–	–	–	–	–	–
LIVESTRONG FB Option Tool	–	✓	✓	✓	✓	–	✓	✓	✓	–	✓	✓	✓	✓	–	✓
Health Educational Materials (printable handouts)																
ASRM Fact Sheet	–	✓	✓	✓	–	–	–	–	–	–	–	–	–	–	–	–
Breast Cancer Care Booklet	✓	✓	✓	✓	✓	–	–	–	✓	✓	✓	✓	–	–	–	✓
CCA Booklet	✓	✓	✓	✓	✓	✓	–	✓	–	✓	✓	✓	✓	✓	✓	✓
Cancer.net	–	✓	✓	✓	✓	–	–	–	–	–	–	–	–	–	–	–
CancerCare Fact Sheet	–	✓	✓	✓	–	–	–	–	–	–	–	–	–	–	–	–
Fertile Future Brochure	–	✓	✓	✓	–	✓	✓	✓	✓	–	✓	✓	✓	✓	–	✓
LIVESTRONG Booklet	–	✓	✓	✓	✓	–	✓	✓	✓	–	✓	✓	✓	✓	–	–
LLSC Fertility Facts	–	✓	✓	✓	✓	✓	–	–	–	✓	✓	✓	✓	–	–	–
Save My Fertility	–	✓	✓	✓	✓	✓	✓	–	–	–	–	–	–	–	–	–
UHN – PMH Pamphlet	✓	✓	✓	–	–	–	–	–	–	–	–	–	–	–	–	–
Health Educational Materials (printable website sections dedicated to oncofertility)																
American Cancer Society	–	✓	✓	✓	✓	✓	–	✓	–	–	✓	✓	✓	✓	–	✓
BreastCancer.org	–	✓	✓	✓	✓	–	–	–	–	–	✓	–	✓	✓	–	–
CancerPoints	–	✓	✓	✓	–	–	–	–	–	–	–	–	✓	✓	–	✓
Canadian Cancer Society (CCS)	–	✓	✓	✓	✓	–	–	✓	✓	–	✓	✓	✓	✓	–	–
Cleveland Clinic	–	✓	✓	✓	✓	–	–	–	–	–	–	–	✓	–	–	–

(Continue on following page)

Multimedia Appendix 3. Fertility and parenthood options in the oncofertility decision aids and health education materials (continued)

Resource Name	Fertility Options Before Treatment									Parenthood Options After Treatment						
	Wait and see	Egg freezing	Embryo freezing	Ovarian tissue freezing	Ovarian suppression	Ovarian transposition	Ovarian shielding	Fertility-sparing surgery	*In vitro* maturation	No more children	Egg donation	Embryo donation	Surrogacy	Adoption	Foster parenting	Natural conception/ Fertility testing
Health Educational Materials (printable website sections dedicated to oncofertility)																
Johns Hopkins Medicine (JHM)	–	✓	✓	✓	✓	✓	–	✓	–	–	✓	–	–	–	–	✓
Mayo Clinic	–	✓	✓	✓	✓	✓	✓	✓	–	–	–	–	–	–	–	✓
MD Anderson Cancer Center	–	✓	✓	✓	✓	✓	–	–	–	–	✓	✓	✓	✓	–	✓
Memorial Sloan Kettering Cancer Center (MSKCC)	✓	✓	✓	✓	✓	✓	–	✓	–	–	✓	✓	–	✓	–	✓
National Comprehensive Cancer Network (NCCN)	–	✓	✓	✓	–	–	–	–	–	–	–	–	✓	–	–	–
National Cancer Institute (NCI)	–	✓	✓	✓	✓	✓	✓	✓	–	–	–	–	–	–	–	–
National Health Service (NHS)	–	✓	✓	✓	–	–	–	–	–	✓	–	–	✓	✓	–	✓
OncoLink	–	✓	✓	✓	✓	✓	–	✓	–	–	–	–	–	–	–	–
WebMD	–	✓	✓	✓	✓	–	–	–	–	–	–	–	–	–	–	–
Young Survival Coalition (YSC)	–	✓	✓	✓	✓	–	–	–	–	–	✓	–	✓	✓	–	✓
Health Educational Materials (non-printable websites dedicated to oncofertility)																
Alliance for FP	–	✓	✓	✓	✓	✓	–	✓	–	–	✓	✓	✓	–	–	✓
Fertile Action	–	✓	✓	✓	✓	–	–	–	–	–	✓	✓	✓	✓	–	✓

Abbreviations: CCA, Cancer Council Australia; FB, family-building; FP, fertility preservation; LLSC, The Leukemia & Lymphoma Society of Canada; PMH, Princess Margaret Hospital; SPOKE, Surgeon and Patient Oncofertility Knowledge Enhancement; UHN, University Health Network
