# Supplementary material for: Oncofertility Decision Support Resources for Women of Reproductive Age: Systematic Review
Source: JMIR Cancer. 2019 Jun 6;5(1):e12593. doi: 10.2196/12593 (PMC6592478; doi:10.2196/12593)
Supplement: Multimedia Appendix 4 [file cancer_v5i1e12593_app4.pdf]

Multimedia Appendix 4. Content and sections in the oncofertility decision aids and health education materials

Resource Name	Background Information				Information on Cancer/Support							Other Sections						
	Explanation of female fertility	Explanation of female infertility	General factors that affect fertility	Role of health care provider	General information about cancer	Types of cancer treatments	Potential fertility outcomes after treatment	Explanation of how treatment impacts fertility	Explanation of treatments impact on pregnancy or lactation	Effects on family, children, and relationships	Psychosocial concerns	VCM	Personal stories	Health care provider directed questions	Sources for more information	Notes section	References	Glossary
Decision Aids																		
Australian Decision Aid	✓	✓	✓	✓	✓	✓	✓	✓	✓	✓	✓	✓	✓	✓	✓	✓	–	✓
Dutch Decision Aid	✓	✓	✓	–	–	✓	–	✓	✓	✓	–	✓	–	✓	✓	–	✓	–
SPOKE Option Grid	–	–	–	–	–	–	–	–	–	–	–	–	–	–	✓	–	–	–
LIVESTRONG FB Option Tool	–	–	–	–	–	✓	–	–	–	–	–	–	–	–	–	–	–	–
Health Educational Materials (printable handouts)																		
ASRM Fact Sheet	–	–	–	–	–	–	–	–	–	–	–	–	–	–	–	–	–	–
Breast Cancer Care Booklet	✓	✓	✓	✓	–	✓	✓	✓	–	✓	✓	–	✓	✓	✓	–	–	–
CCA Booklet	✓	✓	✓	✓	✓	✓	✓	✓	✓	✓	✓	–	✓	✓	✓	–	✓	✓
Cancer.net	–	✓	–	✓	–	–	–	–	–	–	–	–	–	–	–	–	–	–
CancerCare Fact Sheet	–	–	✓	–	–	–	✓	–	–	–	✓	–	–	✓	✓	–	–	–
Fertile Future Brochure	–	✓	–	✓	✓	✓	✓	✓	–	–	–	–	–	–	–	–	✓	✓
LIVESTRONG Booklet	–	✓	✓	–	✓	✓	✓	✓	–	✓	✓	–	✓	✓	✓	–	–	–
LLSC Fertility Facts	–	✓	✓	–	–	–	✓	✓	✓	✓	–	–	–	✓	✓	–	✓	–
Save My Fertility	–	–	–	–	–	–	–	–	–	–	–	–	–	✓	✓	–	✓	✓
UHN – PMH Pamphlet	✓	✓	✓	–	–	–	✓	✓	–	–	–	–	–	–	✓	–	–	–
Health Educational Materials (printable website sections dedicated to oncofertility)																		
American Cancer Society	–	✓	–	–	✓	✓	✓	✓	✓	✓	–	–	–	–	✓	–	✓	–
BreastCancer.org	–	–	✓	–	–	✓	✓	✓	✓	–	–	–	✓	✓	✓	–	–	–

(Continue on following page)

Multimedia Appendix 4. Content and sections in the oncofertility decision aids and health education materials (continued)

Resource Name	Background Information				Information on Cancer/Support							Other Sections						
	Explanation of female fertility	Explanation of female infertility	General factors that affect fertility	Role of health care provider	General information about cancer	Types of cancer treatments	Potential fertility outcomes after treatment	Explanation of how treatment impacts fertility	Explanation of treatments impact on pregnancy or lactation	Effects on family, children, and relationships	Psychosocial concerns	VCM	Personal stories	Health care provider directed questions	Sources for more information	Notes section	References	Glossary
Health Educational Materials (printable website sections dedicated to oncofertility)																		
CancerPoints	–	–	–	–	–	✓	✓	✓	✓	✓	–	–	–	–	✓	–	✓	–
Canadian Cancer Society (CCS)	–	✓	✓	✓	–	✓	✓	✓	✓	–	–	–	✓	✓	✓	–	✓	✓
Cleveland Clinic	–	–	–	–	–	✓	–	✓	–	–	✓	–	–	–	✓	–	–	–
Johns Hopkins Medicine (JHM)	–	–	–	–	–	✓	✓	✓	✓	✓	–	–	–	–	✓	–	–	–
Mayo Clinic	–	–	–	✓	–	–	✓	✓	–	✓	–	–	–	✓	✓	–	–	–
MD Anderson Cancer Center	–	–	–	–	–	✓	✓	✓	✓	–	–	–	–	✓	–	–	–	–
Memorial Sloan Kettering Cancer Center (MSKCC)	✓	–	✓	✓	✓	✓	✓	✓	–	–	✓	–	–	–	✓	–	–	–
National Comprehensive Cancer Network (NCCN)	–	–	–	✓	–	–	✓	✓	–	–	–	–	–	✓	–	–	–	–
National Cancer Institute (NCI)	–	–	✓	–	✓	✓	–	✓	–	–	✓	–	–	✓	✓	–	–	✓
National Health Service (NHS)	✓	–	✓	✓	–	✓	✓	✓	✓	✓	✓	–	–	–	✓	–	–	–
OncoLink	✓	–	✓	–	–	✓	✓	✓	✓	✓	–	–	–	–	–	–	✓	✓
WebMD	–	–	✓	–	✓	✓	✓	✓	–	✓	–	–	–	–	–	–	✓	–
Young Survival Coalition (YSC)	–	–	–	✓	–	✓	–	✓	–	–	–	–	–	–	✓	–	–	–

(Continue on following page)

Multimedia Appendix 4. Content and sections in the oncofertility decision aids and health education materials (continued)

	Background Information				Information on Cancer/Support							Other Sections						
**Resource Name**	Explanation of female fertility	Explanation of female infertility	General factors that affect fertility	Role of health care provider	General information about cancer	Types of cancer treatments	Potential fertility outcomes after treatment	Explanation of how treatment impacts fertility	Explanation of treatments impact on pregnancy or lactation	Effects on family, children, and relationships	Psychosocial concerns	VCM	Personal stories	Health care provider directed questions	Sources for more information	Notes section	References	Glossary
Health Educational Materials (non-printable websites dedicated to oncofertility)																		
Alliance for FP	✓	✓	✓	–	✓	✓	–	✓	–	–	–	–	✓	–	✓	–	✓	–
Fertile Action	✓	✓	✓	–	✓	✓	✓	✓	–	–	–	–	✓	–	✓	–	–	–

Abbreviations: CCA, Cancer Council Australia; FB, family-building; FP, fertility preservation; LLSC, The Leukemia & Lymphoma Society of Canada; PMH, Princess Margaret Hospital; SPOKE, Surgeon and Patient Oncofertility Knowledge Enhancement; UHN, University Health Network; VCM, Values Clarification Method
