# Supplementary material for: Oncofertility Decision Support Resources for Women of Reproductive Age: Systematic Review
Source: JMIR Cancer. 2019 Jun 6;5(1):e12593. doi: 10.2196/12593 (PMC6592478; doi:10.2196/12593)
Supplement: Multimedia Appendix 5 [file cancer_v5i1e12593_app5.pdf]

Multimedia Appendix 5. Quality assessments of the oncofertility decision aids and health education materials

Quality Criteria – International Patient Decision Aid Standards Minimum Standards Instrument	Decision Aids: Australian Decision Aid	Dutch Decision Aid	SPOKE Option Grid	LIVE-STRONG FB Option Tool
**Qualifying**				
Q1 Describes health condition or problem for which index decision is required	✓	✓	–	✓
Q2 Explicitly states decision under consideration (index decision)	✓	✓	✓	✓
Q3 Describes the options available for the index decision	✓	✓	✓	✓
Q4 Describes the positive features of each option	✓	✓	✓	✓
Q5 Describes the negative features of each option	✓	✓	✓	–
Q6 Describes the features of options to help patients imagine the physical, social and/or psychological effects	✓	✓	✓	✓
**Certification**				
C1 Shows positive and negative features of options with equal detail Information	✓	✓	✓	–
C2 Provides information about the funding source used for development	✓	✓	✓	–
C3 Provides citations to the evidence selected	–	✓	–	–
C4 Provides a production or publication date	✓	✓	✓	–
C5 Provides information about update policy	✓	✓	–	–
C6 Provides information about the level of uncertainty around outcome probabilities	✓	✓	✓	✓
CT1 Describes what the test is designed to measure	N/A	N/A	N/A	N/A
CT2 Describes next steps taken if test detects a condition/problem	N/A	N/A	N/A	N/A
CT3 Describes next steps if no condition/problem detected	N/A	N/A	N/A	N/A
CT4 Describes consequences of detection that would not have caused problems if the screen was not done	N/A	N/A	N/A	N/A
**Quality**				
QA1 Development included needs assessment to determine what patients need to make the decision	✓	✓	✓	–
QA2 Development included needs assessment to determine what health professionals need to discuss decision	✓	✓	✓	–
QA3 Development included review by patients not involve in producing the DSI	✓	✓	–	–
QA4 Development included review by professionals not involve in producing the DSI	✓	✓	–	–
QA5 DSI was field tested with patients facing the decision	✓	✓	–	–
QA6 DSI was field tested with practitioners who counsel patients facing the decision	✓	✓	✓	–
QA7 Includes author/developers credentials or qualifications	✓	✓	✓	–
QA8 Evidence that DSI improves match between patient preferences and chosen option	✓	✓	–	–
QA9 Evidence that DSI helps patient improve knowledge about options' features	✓	✓	–	–
QA10 Describes how research evidence was selected/synthesized	–	✓	–	–
QA11 Describes the quality of research evidence used	–	–	–	–
QA12 Provides step by step way to make decision	✓	✓	–	–
QA13 Includes tools to use when discussing options with practitioner	✓	✓	✓	–
QA14 Describes the natural course of the condition	✓	✓	–	✓
QA15 Makes it possible to compare features of available options	✓	✓	✓	✓
QA16 Reports readability levels	–	✓	–	–
QA17 Provides information about outcome probabilities (OPs)	✓	✓	✓	✓
QA18 Specifies reference class of patient for which OPs apply	✓	✓	✓	✓
QA19 Specifies event rates for OPs	✓	✓	✓	✓
QA20 Specifies the time period over which OPs apply	–	–	–	–
QA21 Allows to compare OPs using the same denominator	✓	✓	–	–
QA22 Provides more than one way of viewing probabilities	✓	–	–	–
QA23 Asks patients to consider which positive and negative features matter most to them	✓	✓	–	–
QAT1 Includes information about chances of having a true positive result	N/A	N/A	N/A	N/A
QAT2 Includes information about chances of having a true negative result	N/A	N/A	N/A	N/A
QAT3 Includes information about chances of having a false positive result	N/A	N/A	N/A	N/A
QAT4 Includes information about chances of having a false negative result	N/A	N/A	N/A	N/A
QAT5 Describes the chance the disease is detected with and without use of the test	N/A	N/A	N/A	N/A

Multimedia Appendix 5. Quality assessments of the oncofertility decision aids and health education materials

PEMAT Categories		ASRM Fact Sheet	Breast Cancer Care Booklet	CCA Booklet	Cancer.net	CancerCare Fact Sheet	Fertile Future Brochure	LIVESTRONG Booklet	LLSC Fertility Facts	Save My Fertility	UHN – PMH Pamphlet	American Cancer Society	BreastCancer .org
		**Health Educational Materials (printable handouts and printable website sections dedicated to oncofertility)**											
**UNDERSTANDABILITY**													
**Content**	The material makes its purpose completely evident	–	✓	✓	✓	✓	✓	✓	✓	✓	✓	✓	✓
	The material does not include information or content that distracts from its purpose	✓	✓	✓	✓	✓	✓	✓	✓	✓	✓	✓	✓
**Word Choice & Style**	The material uses common, everyday language.	–	✓	✓	–	–	–	✓	–	✓	✓	✓	✓
	Medical terms are used only to familiarize audience with the terms. When used, medical terms are defined.	–	✓	✓	✓	–	✓	–	–	–	–	✓	✓
	The material uses the active voice	✓	✓	✓	✓	✓	✓	✓	✓	✓	✓	✓	✓
**Use of Numbers**	Numbers appearing in the material are clear and easy to understand	N/A	N/A	✓	N/A	N/A	✓	N/A	N/A	N/A	–	N/A	✓
	The material does not expect the user to perform calculations	✓	✓	✓	✓	✓	✓	✓	✓	✓	✓	✓	✓
**Organization**	The material breaks or "chunks" information into short sections	–	✓	✓	✓	–	✓	✓	✓	–	✓	✓	✓
	The material's sections have informative headers	–	✓	✓	–	✓	✓	✓	✓	–	✓	✓	✓
	The material presents information in a logical sequence	✓	✓	✓	✓	✓	✓	✓	✓	✓	✓	✓	✓
	The material provides a summary	–	–	✓	N/A	–	–	–	✓	N/A	–	–	–
**Layout & Design**	The material uses visual cues to draw attention to key points	–	✓	✓	✓	–	–	✓	✓	✓	✓	✓	–
**Use of Visual Aids**	The material uses visual aids whenever they could make content more easily understood	–	–	–	–	–	–	–	–	–	–	–	–
	The material's visual aids reinforce rather than distract from the content	N/A	✓	✓	✓	✓	–	✓	N/A	✓	–	✓	N/A

Multimedia Appendix 5. Quality assessments of the oncofertility decision aids and health education materials

		Health Educational Materials (printable handouts and printable website sections dedicated to oncofertility)											
**PEMAT Categories**		ASRM Fact Sheet	Breast Cancer Care Booklet	CCA Booklet	Cancer.net	CancerCare Fact Sheet	Fertile Future Brochure	LIVESTRONG Booklet	LLSC Fertility Facts	Save My Fertility	UHN – PMH Pamphlet	American Cancer Society	BreastCancer .org
**Use of Visual Aids**	The material's visual aids have clear titles or captions	N/A	✓	✓	–	–	–	–	N/A	–	–	✓	N/A
	The material uses illustration and photographs that are clear and uncluttered	N/A	✓	✓	✓	✓	✓	✓	N/A	✓	✓	✓	N/A
	The material uses simple tables with short and clear row/column headings	N/A	N/A	✓	N/A	N/A	N/A	✓	N/A	✓	✓	N/A	N/A
**Understandability Score**		4/12	13/15	16/17	10/14	8/15	10/16	12/16	9/12	10/15	11/17	13/15	10/13
**Understandability Score (%)**		33%	87%	94%	71%	53%	63%	75%	75%	67%	65%	87%	77%
**ACTIONABILITY**													
	The material clearly identifies at least one action the user can take	–	✓	✓	✓	✓	✓	✓	✓	✓	✓	✓	✓
	The material addresses the user directly when describing actions	✓	✓	✓	✓	✓	✓	✓	✓	✓	✓	✓	✓
	The material breaks down any action into manageable, explicit steps	–	✓	✓	–	–	–	✓	✓	–	–	–	✓
	The material provides a tangible tool (e.g., menu planners, checklists) whenever it could help the user take action.	–	✓	✓	–	–	–	✓	✓	✓	–	–	✓
	The material provides simple instructions or examples of how to perform calculations	N/A	N/A	N/A	N/A	N/A	N/A	N/A	N/A	N/A	N/A	N/A	N/A
	The material explains how to use the charts, graphs, tables, or diagrams to take actions.	N/A	N/A	N/A	N/A	N/A	N/A	–	N/A	–	✓	N/A	N/A
	The material uses visual aids whenever they could make it easier to act on the instructions.	–	–	–	–	–	–	–	–	–	–	–	–
**Actionability Score**		1/5	4/5	4/5	2/5	2/5	2/5	4/6	4/5	3/6	3/6	2/5	4/5
**Actionability Score (%)**		20%	80%	80%	40%	40%	40%	67%	80%	50%	50%	40%	80%

Multimedia Appendix 5. Quality assessments of the oncofertility decision aids and health education materials

PEMAT Categories		Cancer Points	Canadian Cancer Society (CCS)	Cleveland Clinic	Johns Hopkins Medicine (JHM)	Mayo Clinic	MD Anderson Cancer Center	Memorial Sloan Kettering Cancer Center (MSKCC)	National Comprehensive Cancer Network (NCCN)	National Cancer Institute (NCI)	National Health Service (NHS)	OncoLink	WebMD	Young Survival Coalition (YSC)
		**Health Educational Materials (printable handouts and printable website sections dedicated to oncofertility)**												
**UNDERSTANDABILITY**														
**Content**	The material makes its purpose completely evident	✓	✓	✓	✓	✓	✓	✓	–	✓	✓	✓	✓	✓
	The material does not include information or content that distracts from its purpose.	✓	✓	✓	✓	✓	✓	✓	✓	✓	✓	✓	✓	✓
**Word Choice & Style**	The material uses common, everyday language.	–	✓	✓	–	✓	✓	✓	–	✓	✓	–	✓	✓
	Medical terms are used only to familiarize audience with the terms. When used, medical terms are defined.	–	✓	✓	✓	–	–	✓	–	✓	✓	✓	–	–
	The material uses the active voice	✓	✓	✓	✓	✓	✓	✓	✓	✓	✓	✓	✓	✓
**Use of Numbers**	Numbers appearing in the material are clear and easy to understand	–	N/A	N/A	–	N/A	–	N/A	✓	N/A	N/A	–	–	N/A
	The material does not expect the user to perform calculations	✓	✓	✓	✓	✓	✓	✓	✓	✓	✓	✓	✓	✓
**Organization**	The material breaks or "chunks" information into short sections	✓	✓	✓	✓	✓	✓	✓	✓	✓	✓	✓	✓	✓
	The material's sections have informative headers	✓	✓	✓	✓	✓	✓	✓	–	✓	–	✓	–	✓
	The material presents information in a logical sequence	✓	✓	✓	✓	✓	✓	✓	✓	✓	✓	✓	✓	✓
	The material provides a summary	–	–	–	–	–	–	–	–	–	–	–	–	–
**Layout & Design**	The material uses visual cues to draw attention to key points	–	✓	–	–	✓	–	–	–	–	–	–	–	–
**Use of Visual Aids**	The material uses visual aids whenever they could make content more easily understood	–	–	–	–	–	–	–	–	–	–	–	–	–
	The material's visual aids reinforce rather than distract from the content	N/A	N/A	N/A	N/A	N/A	N/A	✓	–	✓	N/A	N/A	N/A	✓

Multimedia Appendix 5. Quality assessments of the oncofertility decision aids and health education materials

PEMAT Categories		Health Educational Materials (printable handouts and printable website sections dedicated to oncofertility)												
		Cancer Points	Canadian Cancer Society (CCS)	Cleveland Clinic	Johns Hopkins Medicine (JHM)	Mayo Clinic	MD Anderson Cancer Center	Memorial Sloan Kettering Cancer Center (MSKCC)	National Comprehensive Cancer Network (NCCN)	National Cancer Institute (NCI)	National Health Service (NHS)	OncoLink	WebMD	Young Survival Coalition (YSC)
**Use of Visual Aids**	The material's visual aids have clear titles or captions	N/A	N/A	N/A	N/A	N/A	N/A	✓	–	✓	N/A	N/A	N/A	–
	The material uses illustration and photographs that are clear and uncluttered	N/A	N/A	N/A	N/A	N/A	N/A	✓	✓	✓	N/A	N/A	N/A	✓
	The material uses simple tables with short and clear row/column headings	N/A	N/A	N/A	N/A	N/A	N/A	N/A	N/A	N/A	N/A	N/A	N/A	N/A
**Understandability Score**		7/13	10/12	9/12	8/13	9/12	8/13	12/15	7/16	12/15	8/12	8/13	7/13	10/15
**Understandability Score (%)**		54%	83%	75%	62%	75%	62%	80%	44%	80%	67%	62%	55%	67%
**ACTIONABILITY**														
	The material clearly identifies at least one action the user can take	✓	✓	–	–	✓	✓	✓	✓	✓	✓	–	–	✓
	The material addresses the user directly when describing actions	–	✓	N/A	N/A	✓	✓	✓	✓	✓	✓	N/A	N/A	–
	The material breaks down any action into manageable, explicit steps	–	✓	N/A	N/A	–	✓	✓	–	–	–	N/A	N/A	–
	The material provides a tangible tool (e.g., menu planners, checklists) whenever it could help the user take action.	–	✓	–	–	–	✓	✓	✓	–	–	–	–	–
	The material provides simple instructions or examples of how to perform calculations	N/A	N/A	N/A	N/A	N/A	N/A	N/A	N/A	N/A	N/A	N/A	N/A	N/A
	The material explains how to use the charts, graphs, tables, or diagrams to take actions.	N/A	N/A	N/A	N/A	N/A	N/A	N/A	N/A	N/A	N/A	N/A	N/A	N/A
	The material uses visual aids whenever they could make it easier to act on the instructions.	–	–	–	–	–	–	–	–	–	–	–	–	–
**Actionability Score**		1/5	4/5	0/3	0/3	2/5	4/5	4/5	3/5	2/5	2/5	0/3	0/3	1/5
**Actionability Score (%)**		20%	80%	0%	0%	40%	80%	80%	60%	40%	40%	0%	0%	20%

Multimedia Appendix 5. Quality assessments of the oncofertility decision aids and health education materials

Quality Criteria – Seven Quality Domains	Health Educational Materials (non-printable websites dedicated to oncofertility)	
	Alliance for FP	Fertile Action
**Accuracy**		
Based on guidelines, standards of care, literature, textbooks or expert consultation	Yes	Yes
**Technical Elements**		
Ownership of website	Yes	Yes
Disclosure of education, profit, or non-profit interests	Yes	Yes
Clear statement about their objective	Yes	Yes
Audience targeted	Yes	Yes
Transparency of overall sources of funding	Yes	Yes
Compliance with advertising rules	Yes	Yes
Authorship	Yes	No
Date of creation and/or last update of content	No	No
Clear statement about the sources of the content	Yes	No
Creative common license	Yes	Yes
Disclosure of author's affiliation	Yes	No
Disclosure of author's credentials	Yes	No
Information regarding privacy and data protection policy and system for processing of personal data	Yes	Yes
Explicit about data collected from the website visitors and who can access that data	Yes	Yes
Facility to opt in or opt out of subscription service in the form of newsletters or other materials through the website	Yes	No
Message alert about the use of cookies and option to disable it	Yes	Yes
Message alert while leaving a secured website	No	No
Date of last update of the website mentioned, preferably on each page	No	No
Date of technical maintenance of the website	No	No
Email addresses provided as contact information	Yes	Yes
Telephone number provided as contact for customer service	Yes	No
Option for feedback mechanisms	No	Yes
Responsible partnering while providing links to other websites	No	No
Clear statement about editorial review process	No	No
Level of evidence made explicit, such as personal opinion as opposed to peer-reviewed literature	Partial	No
Hierarchy of evidence made clear	No	No
Geographic location of website	Yes	Yes
**Design and Esthetics**		
Good grammar, abbreviations, and acronyms spelled out at first use on each page, and jargon defined	Partial	Partial
Visual appeal of the site, such as alignment and scroll bars	Yes	Yes
Use of subheadings and partitioning long lists to small chunks of 3–5 items	Yes	Partial
Menu with listings, indexes and directional icons	Yes	Yes
Proper layout with illustrations next to the text, visual cueing devices to direct attention to key content, and use of short paragraphs with short sentences	Yes	Partial
Appropriate typography with 12-point type size and text and headlines in upper and lowercases	Yes	Yes
Relevant graphics and images for easy understanding of the content	Yes	No
Inclusion of interactive learning tools	No	No
Illustrations with line drawings familiar to readers	Yes	Yes
Media to communicate the material without autoplay	No	No
Friendly, attractive cover images	Yes	Yes
Information about compatible web browsers	No	No
Quality of visual presentation not compromised when viewed in partial window	Yes	Yes

Multimedia Appendix 5. Quality assessments of the oncofertility decision aids and health education materials

Quality Criteria – Seven Quality Domains	Health Educational Materials (non-printable websites dedicated to oncofertility)	
	Alliance for FP	Fertile Action
**Readability**		
Appropriate sentence construction	Yes	Yes
Use of conversational style, active voice, and simple sentences	Partial	Yes
Road signs to indicate next and previous topics	Yes	Partial
**Usability**		
Functionality to support the content	Yes	Yes
Time to load completely is less than 5 seconds	Yes	Yes
Easy to navigate because of index, table of contents, sitemap, frequently asked questions, and help functions	Yes	Partial
Internal search engine present	Yes	Yes
Option to print or download materials	Partial	No
Restricted access to content by registration requirement or password protection	No	No
Graphic files with "mouse over" indication of graphical content	No	No
Size of large files indicated	No	No
**Accessibility**		
Appropriate color contrasting for font and background color	Yes	Yes
Additional applications such as Adobe Acrobat or Microsoft PowerPoint not required for optimal viewing	Yes	Yes
Easy to find the content	Yes	Yes
Use of preferred language based on target audience	Partial	Partial
Availability for people with disabilities or low-end technology	Partial	Partial
Appropriate images and examples to match the target audience culture	Yes	Yes

Abbreviations: CCA, Cancer Council Australia; FB, family-building; FP, fertility preservation; LLSC, The Leukemia & Lymphoma Society of Canada; PEMAT, The Patient Education Materials Assessment Tool; PMH, Princess Margaret Hospital; SPOKE, Surgeon and Patient Oncofertility Knowledge Enhancement; UHN, University Health Network
