# Supplementary material for: Oncofertility Decision Support Resources for Women of Reproductive Age: Systematic Review
Source: JMIR Cancer. 2019 Jun 6;5(1):e12593. doi: 10.2196/12593 (PMC6592478; doi:10.2196/12593)

Multimedia Appendix 6. Readability level of the oncofertility decision aids and health education materials using the Flesch Kincaid Grade Level

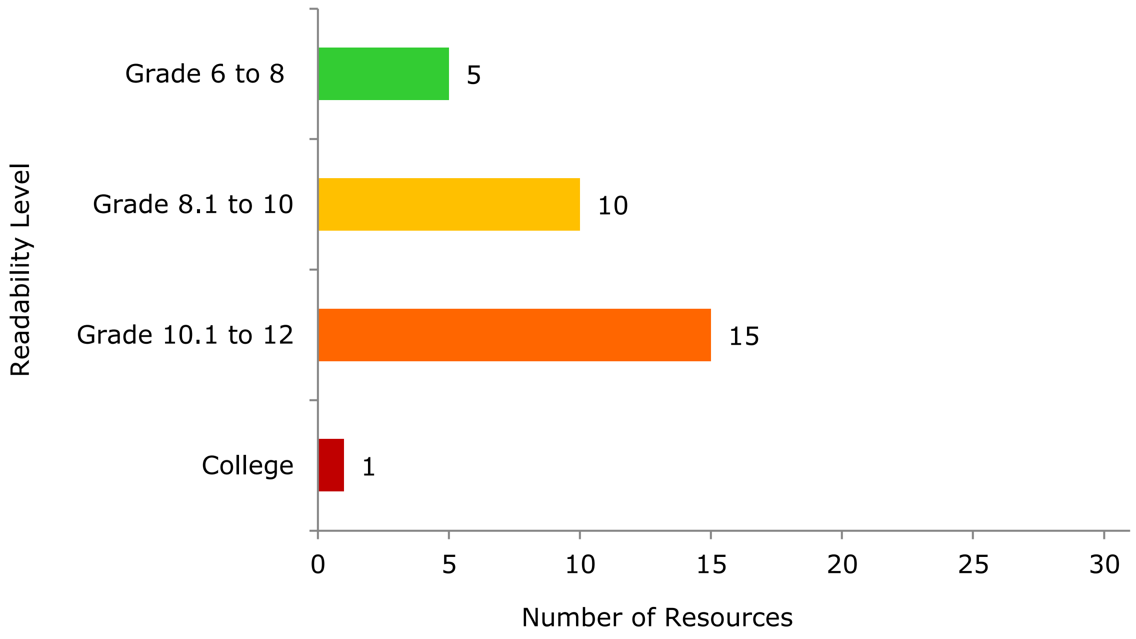

Supplement: Multimedia Appendix 6 [file cancer_v5i1e12593_app6.pdf]
